# Haemorrhage-related maternal mortality in Bangladesh: Levels, trends, time of death, and care-seeking practices based on nationally representative population-based surveys

**DOI:** 10.7189/jogh.13.07001

**Published:** 2023-04-07

**Authors:** Sabrina Jabeen, Abu Bakkar Siddique, Aniqa Tasnim Hossain, Shusmita Khan, M Moinuddin Haider, Tazeen Tahsina, Anisuddin Ahmed, Shafiqul Ameen, Nitai Chakraborty, Quamrun Nahar, Kanta Jamil, Shams El Arifeen, Ahmed Ehsanur Rahman

**Affiliations:** 1 International Centre for Diarrhoeal Disease Research, Bangladesh; 2 Data for Impact, University of North Carolina at Chapel Hill, Chapel Hill, North Carolina, USA; 3 Independent consultant

## Abstract

**Background:**

Haemorrhage is a major cause of maternal deaths globally, most of which are preventable and predominantly happen in low and middle-income countries, including Bangladesh. We examine the current levels, trends, time of death, and care-seeking practices for haemorrhage-related maternal deaths in Bangladesh.

**Methods:**

We conducted a secondary analysis with data from the nationally representative 2001, 2010, and 2016 Bangladesh Maternal Mortality Surveys (BMMS). The cause of death information was collected through verbal autopsy (VA) interviews using a country-adapted version of the standard World Health Organization VA questionnaire. Trained physicians reviewed the VA questionnaire and assigned the cause of death using the International Classification of Diseases (ICD) codes.

**Results:**

Haemorrhage accounted for 31% (95% confidence interval (CI) = 24-38) of all maternal deaths in 2016 BMMS, which was 31% (95% CI = 25-41) in 2010 BMMS and 29% (95% CI = 23-36) in 2001 BMMS. The haemorrhage-specific mortality rate remained unchanged between 2010 BMMS (60 per 100 000 live births, uncertainty range (UR) = 37-82) and 2016 BMMS (53 per 100 000 live births, UR = 36-71). Around 70% of haemorrhage-related maternal deaths took place within 24 hours of delivery. Of those who died, 24% did not seek health care outside the home and 15% sought care from more than three places. Approximately two-thirds of the mothers who died due to haemorrhage gave birth at home.

**Conclusions:**

Postpartum haemorrhage remains the primary cause of maternal mortality in Bangladesh. To reduce these preventable deaths, the Government of Bangladesh and stakeholders should take steps to ensure community awareness about care-seeking during delivery.

Maternal haemorrhage is defined as bleeding from the genital tract during pregnancy (antepartum), birth (intrapartum), or within 42 days after birth (postpartum). Placenta previa or placental abruption causes more than half of the antepartum haemorrhage (APH) cases, while postpartum haemorrhage (PPH) is caused mainly by retained placenta [1,2]. APH and PPH are the leading causes of maternal mortality globally, especially in South Asia and Africa [2-5]. For countries with a high burden of haemorrhage-related maternal deaths, achieving the ambitious sustainable development goal (SDG) target of reducing the maternal mortality ratio (MMR) to less than 70 per 100 000 live births by 2030 will depend on preventing these deaths through early identification, appropriate care-seeking, as well as timely and correct management [4,6].

Bangladesh declared its commitment to achieving the targets set by the 2030 SDG and the Ending Preventable Maternal Mortality roadmap [7,8]. Although the country achieved impressive gains in maternal health during the first decade of the 21st century, the decline in MMR since 2010 indicates apparent stalling [9,10]. The Government of Bangladesh must renew its strategic focus and revisit the program planning to prioritise interventions targeting the prevention and management of major causes of death, including APH and PPH. This will require a critical understanding of the status and bottlenecks related to awareness, access, care-seeking practices, as well as provision and quality of care. Consequently, we aimed to identify the key evidence gaps and present the current level and trends in haemorrhage-related maternal mortality in Bangladesh from the nationally representative Bangladesh Maternal Mortality Survey data. We also wanted to explore care-seeking practices before death, the market share of the public and private sectors, and real-life stories to determine the root causes of haemorrhage-related maternal death related to care-seeking practices and gaps in the healthcare systems.

## METHODS

We performed a secondary analysis of publicly available data from 2001, 2010, and 2016 Bangladesh Maternal Mortality Surveys (BMMS), which were three population-based surveys conducted on a nationally representative sample of households [9,11,12]. Trained data collectors conducted verbal autopsy (VA) interviews for deceased women (13-49 years) of the household using the standard World Health Organization (WHO) VA tool adapted for Bangladesh [13]. Specially trained physicians reviewed the VA forms and assigned a cause of death using the International Classification of Disease (ICD) codes [14]. We grouped similar ICD codes related to obstetric haemorrhage and presented them as a broad category (Table S1 in the **Online Supplementary Document**). More details on the study design, sample size, sampling, and cause of death assignment process are available elsewhere [9,10,12].

### Analysis plan

We used the statistical software package Stata version 14 for data analysis [15]. We adopted descriptive statistics to report the haemorrhage-specific mortality fraction (presented as the percentage of total maternal deaths) and haemorrhage-related maternal mortality ratio (MMR) (presented per 100 000 live births) with 95% confidence intervals (CI) for 2001, 2010, and 2016 BMMS. We also presented the haemorrhage-related MMR stratified by several background, regional, and socioeconomic characteristics to explore potential determinants and changes across the three BMMS.

We estimated the total number of haemorrhage-related maternal deaths per year using the total population reported in the 2011 national census report, the average annual growth rate reported by the World Bank to project the population in 2016, the crude birth rate reported in the 2016 BMMS to estimate the number of live births in 2016, and the haemorrhage-related mortality ratio based on 2016 BMMS data [10,16,17]. We calculated the uncertainty range (UR) using the CI obtained for the haemorrhage-related MMR. The detailed calculations are presented in Table S2 in the **Online Supplementary Document**.

We conducted the analyses on the timing and place of death and care-seeking before death using the 2016 BMMS data. We presented the timing of death by three broad categories: during pregnancy (first trimester, second trimester, third trimester), during delivery (after labour pain but before birth), and after delivery (day 0, day 1, day 2, days 3-6, days 7-42, and ≥43 days). We categorised the place of death as home, health facility (public or private), and in-transit. In-transit deaths refer to deaths that occurred while going from home to a health facility, from one facility to another, or from a facility to home. We also presented the care-seeking patterns with descriptive statistics. Public facilities included district hospitals, maternal and child welfare centres, upazila health complexes, and union health and family welfare centres. Private facilities included private hospitals and clinics.

Lastly, we reviewed the open-ended questions in the VA forms and conducted narrative synthesis to explain patterns identified in the care-seeking practices before the haemorrhage-related maternal deaths.

### Ethical approval

We used publicly available data from the National Institute of Population Research and Training (NIPORT), Bangladesh, and the Monitoring and Evaluation to Assess Use Results (MEASURE Evaluation) project [18,19]. We also obtained approval from MEASURE Evaluation for the secondary analysis.

## RESULTS

### Quantitative

**Figure 1:**
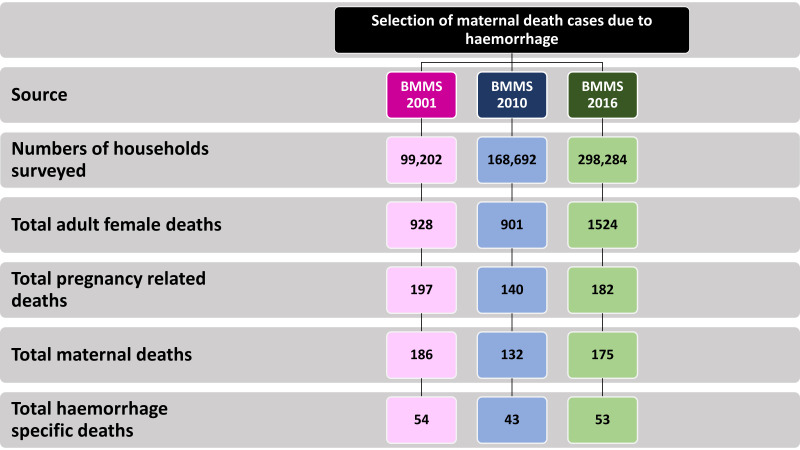
Number of haemorrhage-specific maternal deaths identified in 2001, 2010, and 2016 BMMS.

The number of haemorrhage-related maternal deaths identified from 2001, 2010 and 2016 BMMS are presented in **Figure 1**. The number of live births was 53 805 in 2001 BMMS, 64 529 in 2010 BMMS, and 99 475 in 2016 BMMS. Among these, there were 493 maternal deaths due to haemorrhage, 54 of which occurred in 2001, 43 in 2010, and 53 in 2016.

Haemorrhage-related deaths are still present in rural Bangladesh. Table S3 in the **Online Supplementary Document** presents the background characteristics of the women who died due to haemorrhage-related complications during their most recent pregnancies, births, or postpartum period. Almost two-thirds of the deaths in the 2016 BMMS occurred in rural areas. Around half were in families with lower socioeconomic conditions.

#### Maternal death due to haemorrhage across the survey years

**Figure 2:**
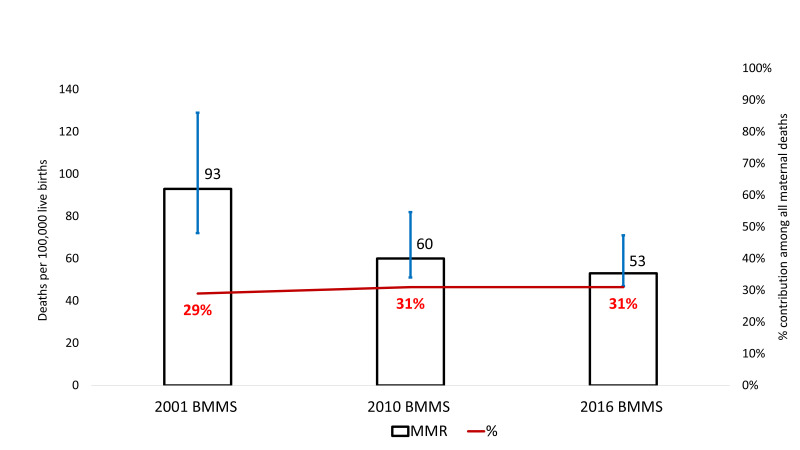
Levels and trends in haemorrhage-specific maternal deaths in Bangladesh, presented in the number of deaths per 100 000 live births and percentage contribution of all maternal deaths.

There has been no substantial progress in reducing maternal death due to haemorrhage across the survey years. **Figure 2** shows the burden of haemorrhage-related maternal deaths in 2001, 2010, and 2016 BMMS. The primary axis indicates the number of haemorrhage-related MMR per 100 000 live births, and the secondary axis presents the percentage of all maternal deaths attributable to haemorrhage-related complications. The haemorrhage-related MMR decreased from 93 (UR = 57 129) per 100 000 live births in the 2001 BMMS to 60 (UR = 37, 82) per 100 000 live births in the 2010 BMMS. However, the decline reached an apparent stalling as it was 53 (UR = 36,71) per 100 000 live births in the 2016 BMMS. In all three BMMS, haemorrhage accounted for around one-third of all maternal deaths.

#### The sociodemographic characteristics of the mothers who died due to haemorrhage changed across the three survey years

**Figure 3:**
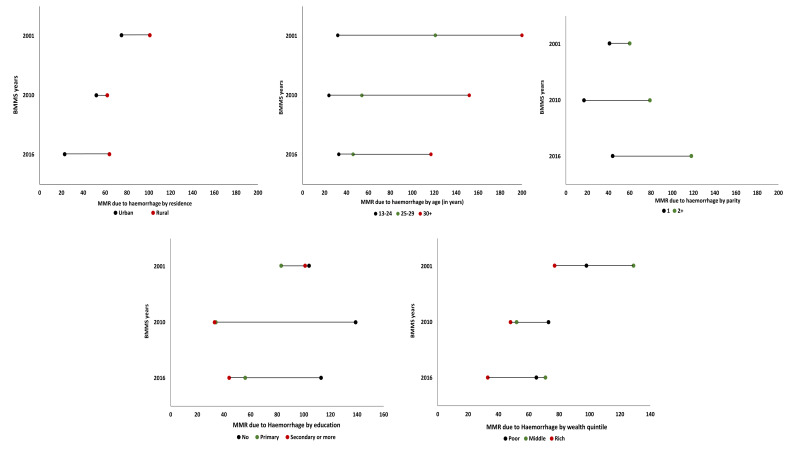
Haemorrhage-related MMR by background characteristics and BMMS rounds, presented in the number of deaths per 100 000 live births.

The sociodemographic characteristics of the mothers who died due to haemorrhage changed across the three survey years. Haemorrhage-related MMR by background characteristics (**Figure 3**) based on 2001, 2010, and 2016 BMMS shows absolute differences in haemorrhage-related MMR between rural and urban areas and between women with no education and primary/early education increased in the 2016 BMMS compared with the 2001 and 2010 BMMS. The absolute difference between the poorer and richer families did not change substantially across the three surveys. However, from the narrative synthesis, we found the impact of poverty on maternal death. By contrast, there was an indication that the absolute difference between the primi- and multi- (three or more) parous women declined during the reporting period. Table S4 in the **Online Supplementary Document** provides additional details, with 95% CI.

#### Estimated deaths due to haemorrhage

**Figure 4:**
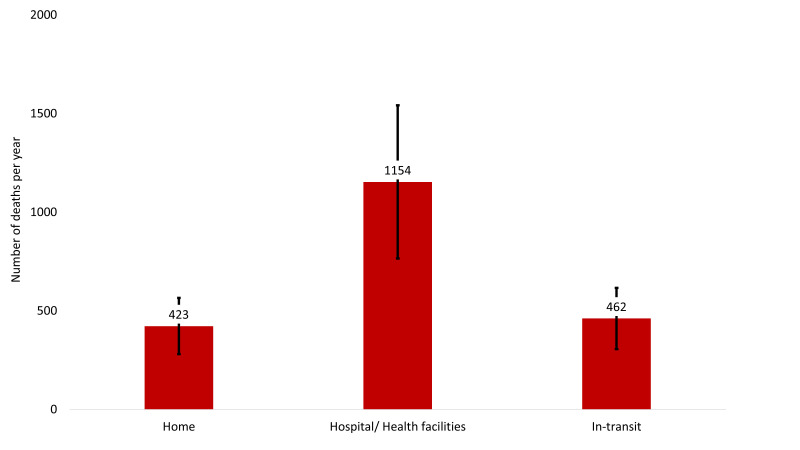
Estimated number of haemorrhage-specific maternal deaths per year in Bangladesh, presented in numbers.

The maximum estimated deaths due to haemorrhage were in hospitals or health facilities. The estimated number of haemorrhage-related maternal deaths per year in Bangladesh is presented in **Figure 4**. Each year, approximately 2039 (UR = 1352, 2725) women die due to haemorrhage-related complications during pregnancy, birth, and in the postpartum period, of which 423 (UR = 281, 566) died at home, 1154 (UR = 765, 1543) in hospitals or health facilities, and 462 (UR = 306, 617) in-transit.

#### Timing of maternal death

**Figure 5:**
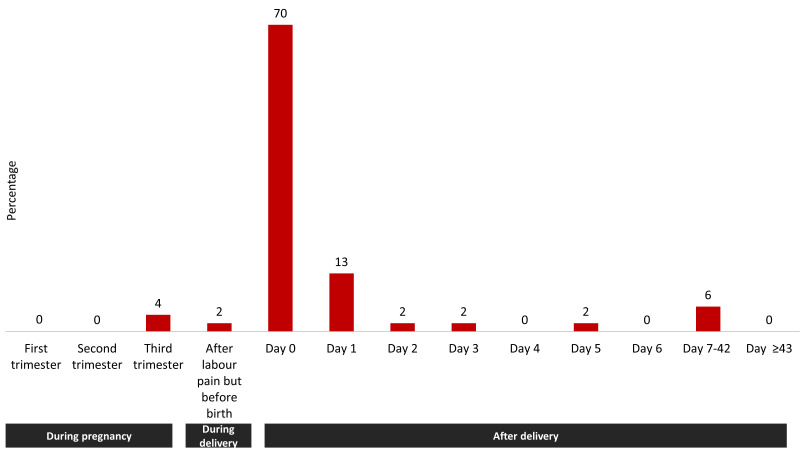
Timing of haemorrhage-specific maternal deaths, presented in percentage distribution (n = 53).

We lost most of the mothers due to haemorrhage on the first day of birth. From the timing of haemorrhage-related maternal deaths (**Figure 5**), we found that 6% (n = 3) of the deaths took place before delivery. Around 70% (n = 37) of the deaths occurred within 24 hours of delivery (day = 0). Another 13% (n = 7) happened between 24 and 48 hours of delivery (day 1).

#### Place of birth of the baby and the place of haemorrhage-specific death of the mother

**Figure 6:**
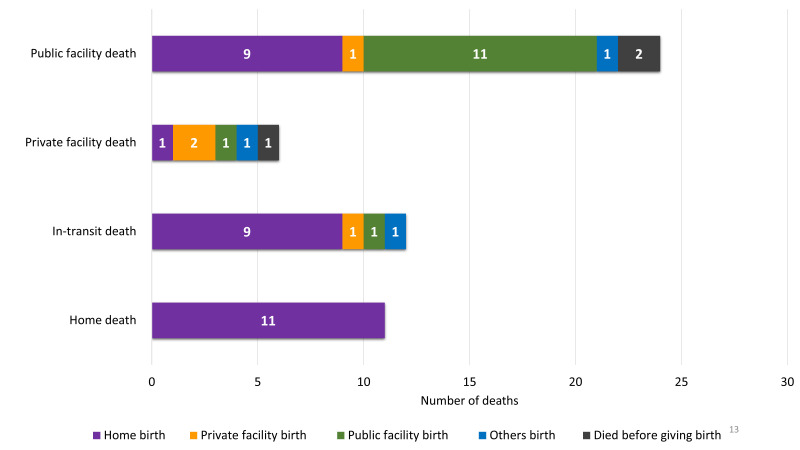
Place of haemorrhage-specific maternal deaths by place of birth, presented in numbers (n = 53).

The baby’s birthplace and the mother’s place of haemorrhage-specific death were not the same for all cases. We summarized the place of maternal deaths due to haemorrhage-related complications by place of birth (**Figure 6**). Other places of birth included the birth of the babies during in-transit. Around one-fifth (n = 11) of the women died at home and another one-fifth (n = 12) died in transit. Around half (n = 24) died in a public facility, and very few (n = 6) died in a private facility. Of the haemorrhage-specific maternal deaths that occurred at home, all gave birth at home. Of the haemorrhage-specific deaths that took place during transit, nine gave birth at home, one in a public facility, and one in a private facility. Of the deaths in a public facility, nine gave birth at home, one in a private facility, and 11 in a public facility. The detailed calculations are presented in Table S5 in the **Online Supplementary Document**.

#### Care-seeking from multiple places during the last illness

**Figure 7:**
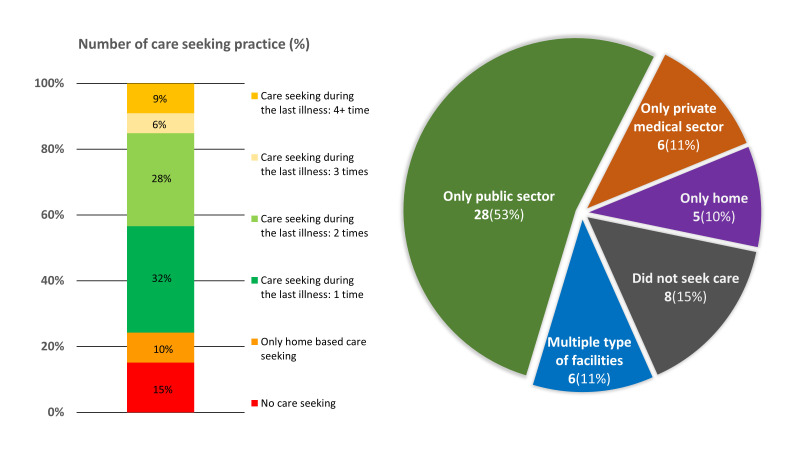
Place of care seeking among maternal deaths due to haemorrhage-related complications, presented in percentage distribution (n = 53).

Care-seeking from multiple places during the last illness was evident from the care-seeking of women who died due to haemorrhage-related complications, the market share of the public and private sectors in care-seeking, and where they sought care from (**Figure 7**). Approximately 15% (n = 8) of the women did not seek care at all, and another 10% (n = 5) only sought home-based care for obstetric haemorrhage. Around one-third (n = 17) sought care once and another 15% (n = 8) had to seek care three or more times. More than half (53%) of the women sought care only from the public sector, whereas 11% (n = 6) sought care only from the private sector. Approximately 11% (n = 6) were taken to multiple types of providers or places.

#### Mapping exercise for the individual case’s care-seeking during death

**Figure 8:**
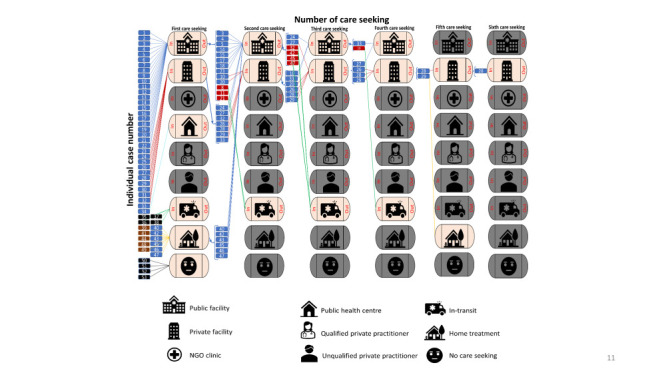
Sequence of care-seeking practices among women who died due to haemorrhage-related complications during pregnancy, birth, and in the postpartum period, presented in numbers (n = 53).

We performed a mapping exercise for the individual case’s care-seeking during death which indicates substantial shuttling. **Figure 8** illustrates the sequence of care-seeking by the individual woman who died due to haemorrhage-related complications during pregnancy, birth, and in the postpartum period. Four women (case numbers 35, 36, 37, and 38) started care-seeking outside the home but died before reaching any facility/provider. Eleven women (case numbers 39, 40, 41, 42, 43, 44, 45, 46, 47, 48, and 49) started with home-based care seeking, of which six women (case numbers 40, 42, 43, 45, 46, and 47) sought care from other facility-based providers during their second care seeking. Twenty-three women initially sought care from public facilities, of which ten went to another facility for the second care-seeking (eight to another public facility and two to a private facility). Ten women initially sought care from private facilities, of which seven went to another facility for the second care-seeking (two to a public facility and five to another private facility). One woman (case number 28) sought care six times from private facilities. Another woman (case number 29) sought care five times from private facilities and afterwards sought home-based care before death. One woman sought care three times from private facilities and afterwards sought care from a public facility. One woman (case number 27) started her care seeking from a private facility, then went to a public facility, then to another private facility, and lastly to a public facility.

### Qualitative

The BMMS questionnaire contained an open-ended section for the respondent to briefly describe the contexts and scenarios that led to death. From the narrative synthesis of cases, we presented two case studies (**Box 1** and **Box 2**) revealing some of the root causes. Additional narrative syntheses of cases from different contexts are presented in the **Online Supplementary Document**

Box 1 **Emergency transportation**

Emergency transportation:

33-year-old Runa (pseudonym) was pregnant with her 6^th^ child and lived in a riverine village with her husband and in-laws. She took regular antenatal care from a nearby facility. During the last ANC, Runa was informed by the service provider that everything around her pregnancy was fine, and she was all good for normal vaginal birth. At her full term, Runa gave birth to a baby boy, with the assistance of a Dai. Everything went well! However, right after Runa’s placenta was delivered, she started to have profuse vaginal bleeding. The Dai tried to control. But nothing was working. After some time, Runa started to have acute chest pain and the bleeding continued. The Dai asked Runa’s family to rush her to a facility. So, her family put blood-soaked Runa in a three-wheeler and rushed to the riverbank. Then they shifted Runa on the boat. But she died on the boat blood soaked.

Box 2 **Shuttling**

Shuttling:

A 20-year-old Maya (pseudonym) was pregnant with her first child. Who used to live in an urban area with her husband and family. Maya came from an educated, middleclass home. Maya took monthly ANCs and the physician told that everything around her pregnancy was just fine. However, since Maya had a history of Asthma, her family decided that they will opt for elective cesarean section. Almost four weeks before her elective cesarean section date, Maya had an asthma attack. The family rushed her to a private clinic. There Maya was kept for 3 days, given oxygen, and managed somehow. And then the doctor referred Maya to the district level public facility. There an emergency c-section was performed and Maya started having excessive vaginal bleeding. And that is how Maya spent 8 long days! With little or no management. And then she was referred to a private medical college hospital. Where she received two bags of blood Maya bleed for two more days, before developing multiple complications. Later, Maya was moved into the intensive care unit. In the ICU, Maya’s heart, lung, kidney gave up. Maya died.

## DISCUSSION

Haemorrhage is the primary cause of maternal deaths in most low and middle-income countries, especially in low-resource settings, including Bangladesh [10,20]. The prevention of haemorrhage-related maternal deaths demands revisiting and reshaping existing policies and strategies addressing the existing bottlenecks and challenges. In this paper, we report that haemorrhage-related complications are the major contributors to overall maternal deaths in the country. After a rapid decline in the first decade of this millennium, haemorrhage-related MMR has not decreased substantially since 2010 and is currently prevailing at a high rate, with wide geographical and wealth-related inequities. More than half of haemorrhage-related maternal deaths happen during or immediately after birth. Most haemorrhage-related maternal deaths came in contact with public facilities before death. There are also major gaps in care-seeking practices, including emergency referral and transportation systems.

We observed that after a rapid decline in the first decade of this millennium with an average annual rate of reduction of 5%, the rate of decline of haemorrhage-related MMR was reduced to only 2.1% since 2010, and is currently prevailing at a high rate, with vast geographical and wealth-related inequities in Bangladesh [9,10]. A similar type of stalling was observed in the reduction of under-five and neonatal mortality rates in Bangladesh [21]. There was much initial enthusiasm among policymakers, program managers, and frontline healthcare providers after the Millennium Development Goals were set, resulting in the development of a new Maternal Health Strategy and the introduction of various maternal health-specific and related programmes and initiatives [22]. With the initial enthusiasm and program interest, Bangladesh may have achieved some more accessible goals related to obstetric haemorrhage prevention and management, but then faced difficulties in sustaining the momentum for relatively more complicated cases due to diminishing attention over time.

We found that APH and PPH account for around one-third of deaths in Bangladesh. This is not unique to Bangladesh as worldwide, 27%, and in the Southwestern Asia region, 30% of maternal deaths take place due to haemorrhage [23]. Gaps in birth preparedness and complication readiness (BPCR), safe birth practice, identification of high-risk pregnancies during antenatal care, structured referral and the emergency transportation system, and facility readiness for managing haemorrhage-related complications and emergencies can potentially explain the high proportion of haemorrhage-related maternal deaths in Bangladesh [24].

### Gaps in birth preparedness and complication readiness (BPCR)

BPCR promotes awareness of danger signs and identification of potential blood donors and arrangement of emergency transportation in advance, which is fundamental for preventing haemorrhage-related maternal deaths [23,25,26]. Unfortunately, there are critical gaps in BPCR among pregnant women and their family members in Bangladesh [25]. A community-based study in Bangladesh reported that only 24% of the women were well-prepared for birth, and around 52% in rural areas in Maharashtra, India [26,27]. Gaps in BPRC among women and family members in Bangladesh may explain the delays in care-seeking practices, which are presented in some of the case studies.

### Gaps in safe birth practice with skilled attendants

Although the coverage of facility births has increased substantially in the past two decades, almost half of the births in rural Bangladesh still happen at home, and almost all of them are conducted by untrained traditional birth attendants [11,28]. We found that around 60% of haemorrhage-related maternal deaths in Bangladesh took place while giving birth at home, almost exclusively with untrained traditional birth attendants (TBA), also locally known as Dai. The narrative synthesis of case studies also exposed the apparent lack of knowledge and skills of untrained traditional birth attendants and unveiled some of the malpractices directly contributing to the demise of the women. A study on 28 social autopsy cases of maternal death in rural areas attributed the delay in decision-making to seek care to the deficiency of TBAs' Knowledge about the management of delivery-related complications [29].

### Gaps in the identification of high-risk pregnancies during antenatal care contacts

Identifying risks during the antenatal period can prevent poor pregnancy outcomes, including haemorrhage-related complications. Antenatal care contacts are one of the best platforms to identify high-risk pregnancies and counsel families about danger signs [30]. Maternal anaemia is one of the common risk factors for APH and PPH-related deaths. Therefore, assessing the blood haemoglobin level during antenatal care contact is crucial for identifying anaemia, which requires appropriate diagnostic provision [30]. In Bangladesh, the current readiness of the health system and the quality of antenatal care are insufficient to identify high-risk pregnancies, including maternal anaemia [28]. A health facility survey conducted in 2017 on a nationally representative sample reported that only 17% of the facilities offering antenatal care services were ready to measure the blood haemoglobin level [11,31]. Only 14% of the women received pregnancy complication-related danger sign counselling during their antenatal care contacts [28]. Similar gaps in the identification of high-risk pregnancies during antenatal care contacts prevail in rural Nepal, as only 62% of women had their blood tests done at least once during their most recent pregnancies [32]. By contrast, data from the maternal monitoring system in eight provinces in China showed that more than 90% of women received a blood test during their antenatal care [33].

### Gaps in structured referral and the emergency transportation system

We found that almost one-fourth of the haemorrhage-related maternal deaths took place in transit, of which the majority gave birth at home. This is indicative of the critical gaps in structured referral and an emergency transportation system in Bangladesh. The most recent version of the Bangladesh Health Facility Survey (BHFS) conducted in 2017 indicated that only 30% of healthcare facilities offering normal delivery services had a functioning ambulance or other vehicles with fuel available on the day of the survey for emergency transportation of patients [11]. Moreover, these vehicles were used only for transferring patients between facilities [22,34,35]. From the narrative synthesis of the case studies, we also observed that the absence of a community-led emergency transportation system or facility-based dedicated ambulance service decisively impacted the minimum window time. Similarly, a qualitative study in rural Odisha, India, found difficulties in seeking care during pregnancy and delivery due to the lack of a public transport system [36].

### Gaps in facility readiness and functionality to manage complications and emergencies

We found that, out of all haemorrhage-related maternal deaths in Bangladesh, more than 40% gave birth in a health facility. A significant proportion of these deaths, primarily due to PPH, could be prevented by the WHO-recommended active management of the third stage of labour (AMTSL) [11]. In Bangladesh, only 40% of the healthcare providers responsible for attending births at health facilities have ever received training in AMTSL [28]. Moreover, there are major issues with the effective availability of uterotonics by maintaining the recommended cold chain, such as oxytocin in health facilities offering normal delivery services [37]. Unfortunately, very few facilities in Bangladesh could maintain the supply and availability of oxytocin per the recommended cold chain, which can substantially increase the risk of PPH and compromise the effectiveness of clinical management [38]. A multi-country qualitative study conducted in India and Myanmar among policymakers, health care providers, pharmacists, and supply chain experts reported inadequate knowledge and awareness about oxytocin's heat sensitivity and the necessity of cold storage [39].

Effective management of severe APH and PPH might require manual removal of placenta and blood transfusion [11]. Among nine comprehensive emergency obstetric and neonatal care (CEmONC) signal functions, three are directly related to haemorrhage management (i.e., use of uterotonic (oxytocin), manual removal of the placenta, and blood transfusion) [40]. Only 57% of the facilities offering normal delivery services reported using oxytocin as uterotonic in the past three months, with 64% for manual removal of the placenta and 16% for blood transfusion [11]. Unfortunately, only 8% of all facilities offering normal delivery services could perform blood grouping and cross-matching [11]. In emergencies, especially during obstetric haemorrhage, healthcare providers had to depend on external sources to arrange blood, which usually takes time and exponentially exposes women to a higher risk of death [41].

### Role of public and private sectors during complications and medical emergencies

We also observed that, out of all haemorrhage-related maternal deaths in Bangladesh, more than half sought care from public health facilities only, while only around 10% sought care from private health facilities. Moreover, around two-thirds of all haemorrhage-related maternal deaths took place in public facilities. This contrasts routine services such as ANC and delivery care, where most now seek care from the private sector [10,28]. The high out-of-pocket expenditure in Bangladesh, particularly during medical emergencies, may explain the preference and dependency on the public sector [42,43]. Sometimes the additional costs result in catastrophic health expenditure, particularly for poorer families [42,44]. The narrative synthesis of case studies we report here also reveals poverty as a significant barrier to care-seeking, especially from the private sector. Moreover, the gaps in readiness and functionality of the private sector to manage complications and medical emergencies may be among other explanatory factors for the observed dependency on the public sector. Per the 2017 BHFS, only 5% of the private facilities offering normal delivery services had emergency management guidelines, 72% had injectable oxytocin, the primary choice of drug for prevention and management of PPH, and 71% performed blood transfusion in the past three months [11]. These suboptimal levels of readiness and functionality of the private sector may result in inappropriate referral of patients to public facilities, often without minimum stabilization. Though we have identified the gaps in the health systems that contributed to haemorrhage-related maternal death, the biological causes are present there as well [45,46]. Even if we think of a healthcare system capable of preventing maximum deaths due to haemorrhage, these biological causes cannot be averted.

### Strengths and limitations

We used data from 2001, 2010, and 2016 BMMS for the secondary analysis [9,10,12]. These surveys were conducted with nationally representative samples and included all administrative divisions of Bangladesh, so we are confident regarding the generalizability of our findings to rural and urban Bangladesh. We used the WHO VA tool, which was adapted to the context of Bangladesh through expert consultations. At least two trained physicians reviewed each form, and the cause of death was assigned based on ICD-10 codes. These measures contributed to ensuring the quality of the data and maintaining international standards and comparability. Moreover, most of the authors of this study were directly involved in the VA component of BMMS, which helped in a better understanding of the data and generating insights from the narrative synthesis.

BMMS was designed to generate a national estimate of MMR. However, we only focused on haemorrhage-related maternal deaths. The sample size was not sufficient to identify the change in haemorrhage-related MMR over time and social determinants and risk factors of haemorrhage-related maternal deaths. We reported the timing of death, care-seeking practice before death, and place of death through descriptive statistics. Due to the small sample size (53 haemorrhage-related deaths), the estimates had wide error margins. Moreover, information regarding the signs and symptoms leading to death, the timing of death, care-seeking practice before death and the place of death were collected from a family member of the deceased woman with a maximum recall of three years. We acknowledge the effect of recall error and recall bias on the relevant information used in our analysis. However, the methodology and maximum recall period used in BMMS are comparable across different rounds and other globally accepted population-level surveys. The narrative synthesis was dependent on the description and details available in the open-ended questions of the VA questionnaire. We acknowledge the issues regarding the accuracy, richness, and standardisation of their narratives. However, we cross-checked the narrative descriptions with quantitative reports to examine internal consistencies before finalising the case studies.

## CONCLUSIONS

Haemorrhage-related MMR is high in Bangladesh and has prevailed as the leading cause of maternal deaths for more than two decades. The strategic focus should be given to improving the readiness, capacity, and functionality of both public and private health facilities for the management of complications and medical emergencies, including APH and PPH. Significant investments should be made in developing a functional referral system which is responsive and accountable. Furthermore, establishing an emergency transportation service that is available, accessible, and affordable to the marginalised and disadvantaged population should be a priority. Lastly, awareness related to maternal danger signs should be improved and complication readiness should be enhanced through strengthening antenatal care contact and counselling.

## Additional material


Online Supplementary Document
