# Supplementary material for: Haemorrhage-related maternal mortality in Bangladesh: Levels, trends, time of death, and care-seeking practices based on nationally representative population-based surveys
Source: J Glob Health. 2023 Apr 7;13:07001. doi: 10.7189/jogh.13.07001 (PMC10080499; doi:10.7189/jogh.13.07001)
Supplement: Online Supplementary Document [file jogh-13-07001-s001.pdf]

ONLINE SUPPLEMENTARY DOCUMENT

Title: Haemorrhage-related maternal mortality in Bangladesh: levels, trends, time of death, and care-seeking practices based on nationally representative population-based surveys

Authors: Sabrina Jabeen[1] (https://orcid.org/0000-0002-0231-2070), Abu Bakkar Siddique[1], Aniqa Tasnim Hossain[1], Shusmita Hossain Khan[2], M Moinuddin Haider[1], Tazeen Tahsina[1], Anisuddin Ahmed[1], Shafiqul Ameen[1], Nitai Chakraborty[2], Quamrun Nahar[1], Kanta Jamil[3], Shams El Arifeen1[1†], Ahmed Ehsanur Rahman1[1†] (https://orcid.org/0000-0001-9216-1079)

Table S1: Summary matrix of ICD-10 codes used to present the cause of death in categories

Broad Category	ICD-10 Code
Maternal haemorrhage	O46.9
	O67.9
	O71.0
	O71.1
	O72
	O72.0

Table S2: Estimated number of maternal deaths due to haemorrhage, by place of birth and death in 2021

		N	%	MMR	95% CI - LB	95% CI - UB
Place of birth	Non-institutional	33	62.3	1269	842	1697
	Institutional	17	32.1	654	434	874
	Died before birth	3	5.7	115	77	154
Place of death	Home	11	20.8	423	281	566
	Hospital/ clinic	30	56.6	1154	765	1543
	In-transit	12	22.6	462	306	617

LB=lower bound; UB=upper bound

Table S3: Background characteristics of the mothers who died of haemorrhage (N=150)

Background characteristics	2001 BMMS		2010 BMMS		2016 BMMS	
	Percent	Number of women	Percent	Number of women	Percent	Number of women
**Age at death**						
15–24	18.5	10	23.3	10	34	18
25–29	24.1	13	23.3	10	24.5	13
30+	57.4	31	53.5	23	41.5	22
**Education**						
No education	57.4	31	51.2	22	17	9
Primary	25.9	14	23.3	10	30.2	16
Secondary+	16.7	9	25.6	11	52.8	28
**Area**						
Rural	87	47	60.5	26	67.6	25
Urban	13	7	39.5	17	32.4	12
**Parity**						
1	11.1	6	11.6	5	20.8	11
2+	88.9	48	88.4	38	79.3	42
**Wealth quintile**						
Poor	46.3	25	55.8	24	47.2	25
Middle	25.9	14	11.6	5	28.3	15
Rich	27.8	15	32.6	14	24.5	13
**Total**	**100**	**54**	**100**	**43**	**100**	**53**

Table S4: MMR, by background characteristics in the 2001 BMMS, 2010 BMMS, and 2016 BMMS

	2001 BMMS			2010 BMMS			2016 BMMS		
Background characteristics	MMR due to haemorrhage (per 100,000)	95% CI		MMR due to haemorrhage (per 100,000)	95% CI		MMR due to haemorrhage (per 100,000)	95% CI	
		Lower bound	Upper bound		Lower bound	Upper bound		Lower bound	Upper bound
**Residence**									
Rural	101	65	137	62	36	88	64	42	87
Urban	75	7	143	52	8	96	23	1	45
**Age in years**									
13–24	32	6	58	24	4	45	33	14	52
25–29	121	47	194	54	12	96	46	15	77
30+	200	110	291	152	74	230	117	60	173
**Parity**									
1	41	16	67	17	0	39	44	4	84
2+	60	37	83	79	47	110	118	76	160
**Education**									
No education	104	55	152	139	70	209	113	29	197
Primary (<=5 years)	83	30	136	34	4	64	56	22	89
Secondary or more (6+ years)	101	33	168	33	8	59	44	24	64
**Wealth quintile**									
Poor	98	51	145	73	35	112	65	35	95
Middle	129	44	213	52	4	100	71	25	116
Rich	77	28	125	48	15	81	33	11	54
**Total**	**97**	**65**	**129**	**60**	**37**	**82**	**53**	**36**	**71**

Table S5: Place of birth vs place of death

Place of birth	N	Place of death				
		Home	In-transit	Public facility	Private facility	Total
		%	%	%	%	%
Home	30	11 (37%)	9 (30%)	9 (30%)	1 (3%)	100
Public	13	0	1 (8%)	11 (85%)	1 (8%)	100
Private	4	0	1 (25%)	1 (25%)	2 (50%)	100
Other	3	0	1 (33%)	1 (33%)	1 (33%)	100
Died before giving birth	3	0	0	2 (67%)	1 (33%)	100
**Total**	**53**	**11 (21%)**	**12 (23%)**	**24 (45%)**	**6 (11%)**	**100**

BOX S1: Untrained birth attendant

Untrained birth attendant:

35-year-old Asma (pseudonym) lived in a urban area and was pregnant for the third time. She had two eventful pregnancies, and her third pregnancy was also full of complications and her major complaint was having palpitations on a regular basis. Despite that, her family decided to go for a home birth and called in a traditional birth attendant (Dai) when she went into the labour which lasted for two days. Right after delivering the placenta with some difficulty – pulling over and over by the Dai, resulted in profuse bleeding. Within a very short time her cloths, the bed, floor, everything became a pool of blood. Her eyes, face, and palms started to pale away and gradually Asma became lethargic. Asma died without any proper care seeking or management.

BOX S2: Poverty

Poverty:

During the last pregnancy – which was her 4th one – Salma (pseudonym) was 34 years old. Throughout her pregnancy she always felt unusually weak, and took ANC from a tertiary-level teaching hospital. She was prescribed some medicines and advised to eat nutritious foods. Thinking about the expenses of the medicines and fearing that nutritious food would make the baby in womb large and will result in having c-section; Salma decided to skip the medicines and food. Salma rather opted to spend the limited money they had on their son's education. During the last ANC, the doctor advised her to undergo a c-section as Salma was not fit for vaginal birth. However, Salma again thought about the expenses of c-section and came back home to home birth. After a week, she gave birth at home with an NGO trained birth attendant and the placenta was not delivered properly. This resulted in excessive vaginal bleeding. Her husband went out to arrange an ambulance, but both Salma and the newborn died before the husband returned.
